# Upregulation of Fatty Acid Transporters is Associated With Tumor Progression in Non-Muscle-Invasive Bladder Cancer

**DOI:** 10.3389/pore.2021.594705

**Published:** 2021-03-30

**Authors:** Hoiseon Jeong, Hwa Eun Oh, Hyesun Kim, Ju-Han Lee, Eung Seok Lee, Young-Sik Kim, Jung-Woo Choi

**Affiliations:** Department of Pathology, Korea University Ansan Hospital, Ansan, Korea

**Keywords:** bladder cancer, Fatty acid, FATP4, CD36, ACSL1

## Abstract

As patients with non-muscle-invasive bladder cancer (NMIBC) show a high degree of heterogeneity in tumor recurrence or progression, many clinicians demand a detailed risk stratification. Although modified fatty acid metabolism in cancer cells is reported to reflect malignant phenotypes such as metastasis, the impact of fatty acid transporters on NMIBC has never been investigated. This study examined the clinicopathologic implications of fatty acid transporters such as fatty acid transport protein 4 (FATP4), cluster of differentiation 36/fatty acid translocase (CD36/FAT), and long chain acyl CoA synthetase 1 (ACSL1) in 286 NMIBC cases. This study revealed that FATP4, CD36, and ACSL1 were overexpressed in 123 (43.0%), 43 (15.0%), and 35 (12.2%) NMIBC cases, respectively. High FATP4 in tumor cells was associated with high grade (*p* = 0.004) and high stage (*p* = 0.039). High CD36 was related to high grade (*p* < 0.001), high stage (*p* = 0.002), and non-papillary growth type (*p* = 0.004). High ACSL1 showed an association with high grade (*p* < 0.001), high stage (*p* = 0.01), non-papillary growth type (*p* = 0.002), and metastasis (*p* = 0.033). High FATP4 was an independent factor predicting short overall survival (OS) (hazard ratio = 3.32; 95% confidence interval, 1.07–10.31; *p* = 0.038). In conclusion, upregulation of FATP4, CD36, and ACSL1 might promote the NMIBC progression and could be exploited in clinical risk stratification and targeted therapy.

## Introduction

Urothelial carcinoma has been known to develop via two distinct pathways, non-muscle-invasive bladder cancer (NMIBC) and muscle-invasive bladder cancer (MIBC) [1]. Both subtypes have unique pathological features and different molecular characteristics. More than 70% of newly diagnosed bladder cancers belong to NMIBC, which is characterized morphologically by frequent papillary structures, and genetically by deletion of chromosome 9 and point mutation of fibroblast growth factor receptor 3 (FGFR3). FGFR3 signaling has been suggested to promote the activation of sterol regulatory element-binding protein 1, a key regulator of lipogenesis [2]. As patients with NMIBC show extreme heterogeneity in terms of recurrence, progression, and survival rates, detailed risk stratification is required for the optimal management of cancer patients.

Unlike normal cells, cancer cells require large amounts of fatty acids to supply signaling molecules, basic cellular structural elements, and sources of metabolic energy such as adenosine triphosphate [3]. Therefore, cancer cells upregulate fatty acids via *de novo* lipogenesis, lipolysis of intracellular lipid droplets, or uptake of fatty acids from the extracellular milieu. *In vitro* and *in vivo* studies have shown that fatty acid composition in bladder cancer cells differs from that in normal urothelial cells, underscoring the altered lipid metabolism in bladder cancer cells [4, 5]. Moreover, overexpression of fatty acid synthase is described as a biomarker for tumor aggressiveness in bladder cancers [6]. Although upregulation of fatty acid synthesis or fatty acid oxidation is reported to be associated with malignant phenotypes such as metastasis or therapeutic resistance, the prognostic effect of fatty acid transporters on human cancers has not been fully investigated [7].

Several candidate proteins have been identified to facilitate the uptake of fatty acids from extracellular sources. An earlier report suggested that co-expression of cluster of differentiation 36/fatty acid translocase (CD36/FAT) with fatty acid transport protein 4 (FATP4) or long chain acyl CoA synthetase 1 (ACSL1) synergistically facilitated fatty acid uptake through active transport of fatty acids across the cell membrane by CD36, and then intracellular trapping of transported fatty acids in the form of acyl-CoA by FATP4 or ACSL1 [8]. CD36 is known to induce tumor progression and metastasis via altered lipid metabolism in various human cancers including bladder cancer [9, 10]. High FATP4 expression has been reported to promote tumor progression in breast and kidney cancers [11, 12]. However, ACSL1 was proposed to function as either an oncogene or tumor-suppressive gene depending on the cancer type [13]. The protein expression and prognostic value of FATP4 and ACSL1 have never been studied in human bladder cancers.

In this study, we examined the protein expression of fatty acid transporters such as FATP4, CD36, and ACSL1 in 286 NMIBC cases by immunohistochemistry. We also evaluated the clinicopathologic implications of FATP4, CD36, and ACSL1 expressions in this NMIBC cohort.

## Materials and Methods

### Tissue Samples

In total, 286 consecutive patients with NMIBC who were at their first diagnosis during the years 1995–2010 were enrolled in the study. Among them, 265 patients were treated with tranurethral resection, while 21 underwent partial or radical cystectomy. The specimens were examined at the Department of Pathology, Korea University Ansan Hospital. In all cases, representative tissues were fixed in 10% buffered formalin, embedded in paraffin, sectioned and stained with hematoxylin and eosin for histological assessment. Tumor stage was ranked according to the guidelines by the American Joint Committee on Cancer, and was classified into low (non-invasive, Ta) and high (invasive, T1) [14]. For the evaluation of muscle invasiveness, we tried to integrate all the available data acquired from patient symptoms, cystoscopy, abdominopelvic computed tomography, and surgical samples. Tumor grade was based on the criteria of the World Health Organization Classification, and included low and high grades [15]. Grossly, tumor growth type was divided into papillary and non-papillary. Recurrence was defined as cystoscopically visible tumors with histologic confirmation. Complete information on the recurrence, metastasis and cause of death was available for all cases. This research was approved by the institutional Ethics Committee of the Korea University Ansan Hospital (IRB No. 2020AS0224). Under the condition of retrospective archival tissue collection and patient data anonymization, our study was exempted from the acquisition of informed consent from patients.

### Tissue Microarray Construction, Immunohistochemistry, and Semi-Quantification

When reviewing the H&E-stained sections from each formalin-fixed, paraffin-embedded block, we searched for the most diagnostic areas. Three representative 1 mm cores obtained from each case were inserted in a grid pattern into a recipient paraffin block using a tissue arrayer (Beecher Instruments Inc., Sun Prairie, WI, United States). Immunohistochemistry was performed using an automated immunostainer (Bond-Max™ Leica Microsystems, Melbourne, Australia), as per the manufacturer’s protocol. Representative tissue sections were deparaffinized and dehydrated, and heat pretreatment was performed for 20 min. After incubation with peroxide for 10 min, the slides were stained with antibodies against FATP4 (clone EPR17319, Abcam, Cambridge, United Kingdom), CD36 (clone D8L9T, Cell Signaling Technology, Danvers, MA, United States), and ACSL1 (clone EPR13499, Abcam, Cambridge, United Kingdom). The working dilution of each antibody was 1:100, 1:400, and 1:200, respectively. After incubation with secondary antibodies at room temperature for 10 min, the sections were developed with 3, 3′-diaminobenzidine, and counterstained with Harris hematoxylin.

Evaluation was performed according to the following immunohistochemical scoring system. Staining intensity was scored as 0 (negative), 1 (weak), 2 (moderate), or 3 (intense). The extent of staining was scored as 0 (negative), 1 (less than 20% of tumor cells stained), 2 (20–50%), 3 (50–80%), or 4 (greater than 80%). The sum of these two scores gave each case a final score of 0–7. A final score of more than 6, 4, and 5 was considered as positive for FATP4, CD36, and ACSL1, respectively. Each cut-off value was determined using receiver operator characteristics curves, which calculate sensitivity and specificity based on tumor grade. For each case, the tissue microarray core showing the highest score was selected for statistical analysis.

### Statistical Analysis

The protein expression of FATP4, CD36, and ACSL1 was compared for patient characteristics using the Chi-square or Fisher’s exact test. The relationship between these fatty acid transporters was evaluated by the bivariate correlation analysis. Overall and recurrence-free survivals were analyzed with Kaplan-Meier curves and tested with the log-rank test. The influence of possible confounding factors was analyzed by a Cox proportional hazards model using a non-stepwize method. Statistical significance was defined as *p* < 0.05. All statistical analyses were performed with SPSS for Windows 10.0 (SPSS Inc., Chicago, IL, United States).

## Results

### Patients

The patients included 254 (88.8%) men and 32 (11.2%) women with an age at diagnosis of 23–97 years (median 68). The cancer was low grade in 144 (50.3%) and high grade in 142 (49.7%) cases; 148 (51.7%) had low stage (Ta), and 138 (48.3%) had high stage (T1). Grossly, tumor growth type was papillary in 221 (77.3%) and non-papillary in 65 (22.7%). Seven patients (2.4%) showed lymphatic invasion and 16 (5.6%) had distant metastases. Local recurrences were observed in 131 (45.8%) patients during their clinical courses. Sixteen patients (5.6%) died of bladder cancer and its complications such as urinary tract infections. The median follow-up period for the population studied was 37 months (range 1–183 months).

### FATP4, CD36, and ACSL1 Expression in NMIBC

**FIGURE 1:**
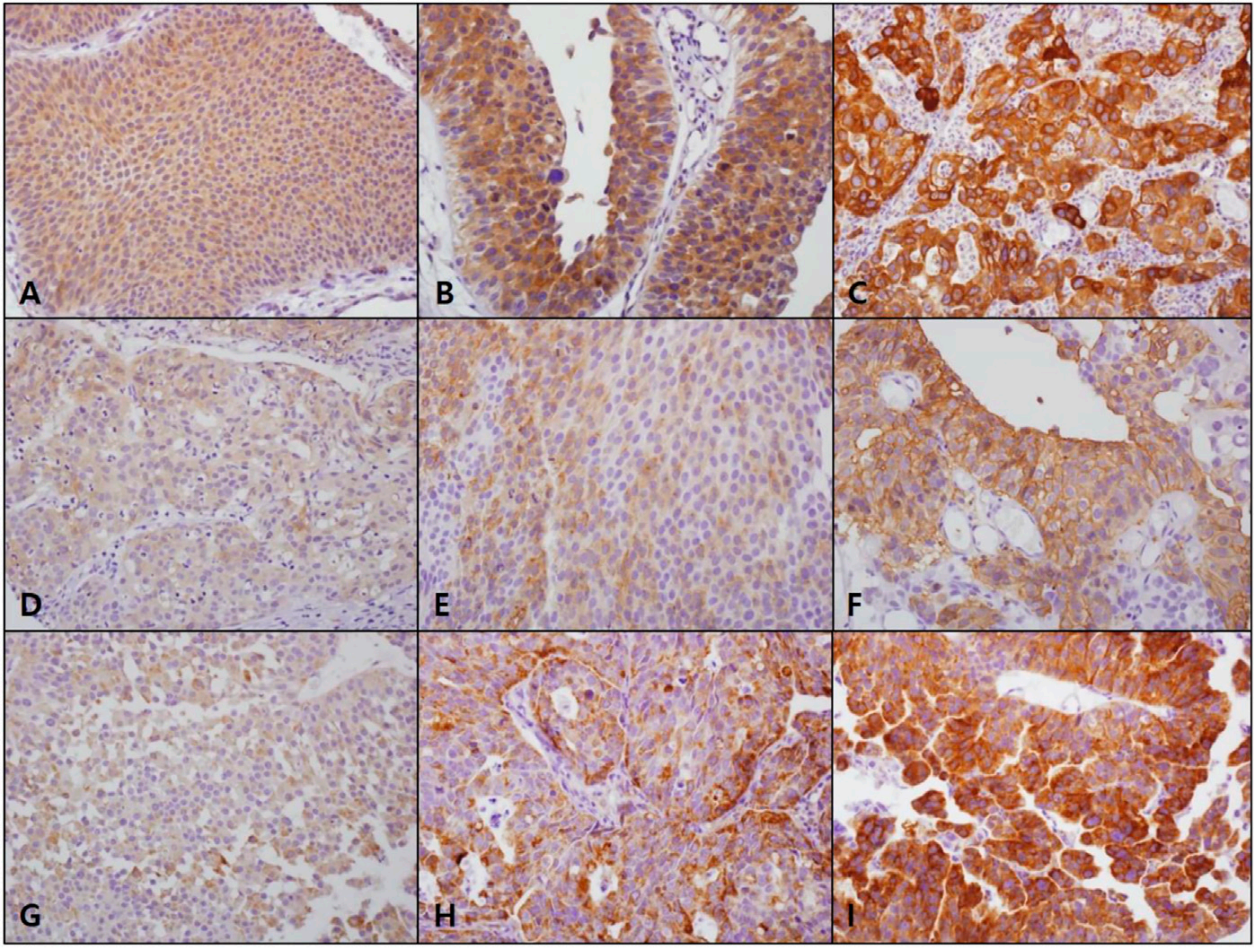
FATP4, CD36, and ACSL1 expression in non-muscle-invasive bladder cancer. FATP4 (A, score 1; B, score 2; C, score 3) and ACSL1 (G, score 1; H, score 2; I, score 3) mostly revealed cytoplasmic staining in bladder cancer cells, while CD36 (D, score 1; E, score 2; F, score 3) was localized to the cell membrane (×400, original magnification).

FATP4, CD36, and ACSL1 overexpression was observed in 123 (43.0%), 43 (15.0%), and 35 (12.2%) cases of NMIBC, respectively (Table 1). FATP4 revealed a mostly cytoplasmic and rare membranous staining in tumor cells, whereas CD36 was mainly expressed in the membrane and rarely in the cytoplasm of bladder cancer cells (Figure 1, and Supplementary Figures S1–S3). CD36 was also detected in vascular endothelial cells. ACSL1 was exclusively localized to the cytoplasm of bladder cancer cells. FATP4, CD36, and ACSL1 were not found in the adjacent normal urothelial cells, whereas the umbrella cells showed occasional positive staining for all these protein markers. High FATP4 expression in tumor cells was associated with high grade (*p* = 0.004) and high stage (*p* = 0.039). High CD36 was related to high grade (*p* < 0.001), high stage (*p* = 0.002), and non-papillary growth type (*p* = 0.004). High ACSL1 showed an association with high grade (*p* < 0.001), high stage (*p* = 0.01), non-papillary growth type (*p* = 0.002), and metastasis (*p* = 0.033). FATP4, CD36, and ACSL1 expression in NMIBC tumor tissues revealed a weak positive correlation among them (FATP4 vs. CD36, Spearman rho = 0.273, *p* < 0.001; FATP4 vs. ACSL1, Spearman rho = 0.378, *p* < 0.001; CD36 vs. ACSL1, Spearman rho = 0.260, *p* < 0.001).

**TABLE 1:** FATP4, CD36, and ACSL1 expression with the clinicopathologic parameters of non-muscle-invasive urothelial carcinoma.

Characteristics		FATP4		*p*	CD36		*p*	ACSL1		*p*
		High (n = 123) No. (%)	Low (n = 163) No. (%)		High (n = 43) No. (%)	Low (n = 243) No. (%)		High (n = 35) No. (%)	Low (n = 251) No. (%)	
Age	Low (<68 years)	59 (48.0)	80 (49.1)	0.852	21 (48.8)	118 (48.6)	0.973	18 (51.4)	121 (48.2)	0.721
	High (≥68 years)	64 (52.0)	83 (50.9)		22 (51.2)	125 (51.4)		17 (48.6)	130 (51.8)	
Sex	Male	106 (86.2)	148 (90.8)	0.220	39 (90.7)	215 (88.5)	0.798	31 (88.6)	223 (88.8)	1.000
	Female	17 (13.8)	15 (9.2)		4 (9.3)	28 (11.5)		4 (11.4)	28 (11.2)	
Grade	Low	50 (40.7)	94 (57.7)	**0.004**	10 (23.3)	134 (55.1)	**<0.001**	5 (14.3)	139 (55.4)	**<0.001**
	High	73 (59.3)	69 (42.3)		33 (76.7)	109 (44.9)		30 (85.7)	112 (44.6)	
T stage	Low (Ta)	55 (44.7)	93 (57.1)	**0.039**	13 (30.2)	135 (55.6)	**0.002**	11 (31.4)	137 (54.6)	**0.010**
	High (T1)	68 (55.3)	70 (42.9)		30 (69.8)	108 (44.4)		24 (68.6)	114 (45.4)	
Growth type	Papillary	91 (74.0)	130 (79.7)	0.249	26 (60.5)	195 (80.2)	**0.004**	20 (57.1)	201 (80.1)	**0.002**
	Non-papillary	32 (26.0)	33 (20.2)		17 (39.5)	48 (19.8)		15 (42.9)	50 (19.9)	
Lymphatic invasion	Absent	121 (98.4)	158 (96.9)	0.703	42 (97.7)	237 (97.5)	1.000	34 (97.1)	245 (97.6)	1.000
	Present	2 (1.6)	5 (3.1)		1 (2.3)	6 (2.5)		1 (2.9)	6 (2.4)	
Metastasis	Absent	115 (93.5)	155 (95.1)	0.609	38 (88.4)	232 (95.5)	0.074	30 (85.7)	240 (95.6)	**0.033**
	Present	8 (6.5)	8 (4.9)		5 (11.6)	11 (4.5)		5 (14.3)	11 (4.4)	
Recurrence	Absent	68 (55.3)	87 (53.4)	0.748	22 (51.2)	133 (54.7)	0.665	19 (54.3)	136 (54.2)	0.991
	Present	55 (44.7)	76 (46.6)		21 (48.8)	110 (45.3)		16 (45.7)	115 (45.8)	

### Prognostic Implications of FATP4, CD36, and ACSL1 Expression in NMIBC

**FIGURE 2:**
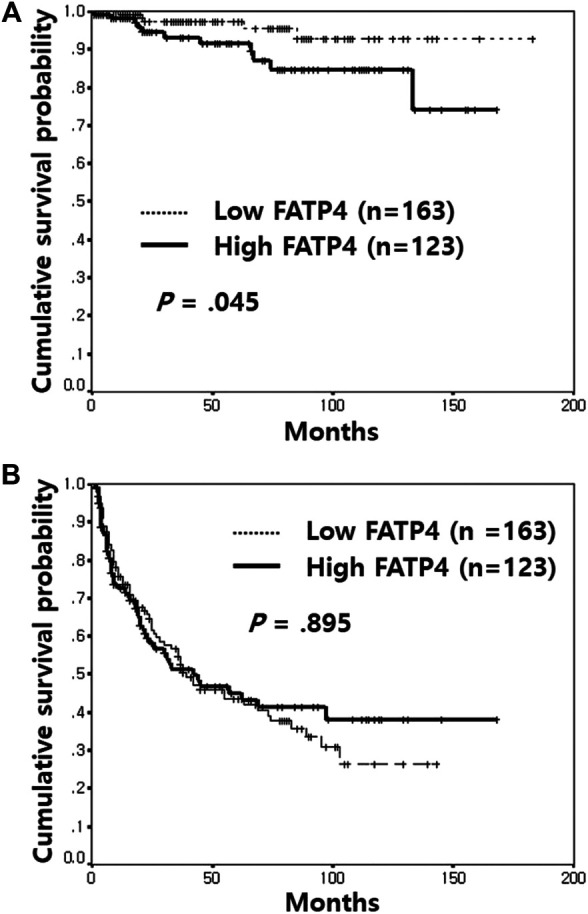
Kaplan-Meier survival analysis of FATP4 expression in non-muscle-invasive bladder cancer. Patients with high FATP4 expression showed a poorer overall survival rate than those with low FATP4 **(A)**. In contrast, high expression of FATP4 was not correlated with recurrence-free survival **(B)**.

In univariate analyses, high grade (*p* = 0.007), high stage (*p* = 0.002), lymphatic invasion (*p* < 0.001), distant metastasis (*p* < 0.001), and high FATP4 (Figure 2A, *p* = 0.045) were associated with poor OS (Table 2). High CD36 and ACSL1 failed to predict overall survival of bladder cancer patients (Supplementary Figures S4A,B). Old age (*p* = 0.044), non-papillary growth type (*p* = 0.008), lymphatic invasion (*p* < 0.001), and distant metastasis (*p* < 0.001) displayed an association with short recurrence-free survival (RFS). However, high expression of FATP4 (Figure 2B), CD36 (Supplementary Figure S4C), and ACSL1 (Supplementary Figure S4D) were not correlated with RFS.

**TABLE 2:** Variables affecting overall and recurrence-free survivals by univariate analyses.

Variable		No	Overall survival		Recurrence-free survival	
			No. of events	*p*	No. of events	*p*
Age	Low (<68 years)	151	7	0.156	62	**0.044**
	High (≥68 years)	135	9		69	
Sex	Male	254	15	0.533	115	0.44
	Female	32	1		16	
Grade	Low	144	3	**0.007**	64	0.279
	High	142	13		67	
Stage	Low (Ta)	148	3	**0.002**	67	0.092
	High (T1)	138	13		64	
Growth type	Papillary	221	10	0.064	96	**0.008**
	Non-papillary	65	6		35	
Lymphatic invasion	Absent	279	13	**<0.001**	126	**<0.001**
	Present	7	3		5	
Metastasis	Absent	270	8	**<0.001**	117	**<0.001**
	Present	16	8		14	
FATP4	Low	163	5	**0.045**	76	0.895
	High	123	11		55	
CD36	Low	243	12	0.246	110	0.611
	High	43	4		21	
ACSL1	Low	251	12	0.09	115	0.933
	High	35	4		16	

In multivariate analyses, high FATP4 (hazard ratio = 3.32; 95% confidence interval, 1.07–10.31; *p* = 0.038), lymphatic invasion (hazard ratio = 13.34; 95% confidence interval, 3.41–52.16; *p* < 0.001), and distant metastasis (hazard ratio = 17.35; 95% confidence interval, 6.02–49.99; *p* < 0.001) were independent factors for poor OS, whereas old age (hazard ratio = 1.44; 95% confidence interval, 1.02–2.04; *p* = 0.039), lymphatic invasion (hazard ratio = 3.40; 95% confidence interval, 1.31–8.82; *p* = 0.012), and distant metastasis (hazard ratio = 2.27; 95% confidence interval, 1.26–4.07; *p* = 0.006) were independent variables for reduced RFS (Table 3).

**TABLE 3:** Poor prognostic factors affecting overall and recurrence-free survival identified by multivariate analyses.

Variables	Unfavorable factor	HR (95% CI)	*p*
Overall survival			
FATP4	High	3.32 (1.07–10.31)	0.038
Lymphatic invasion	Present	13.34 (3.41–52.16)	<0.001
Metastasis	Present	17.35 (6.02–49.99)	<0.001
Recurrence-free survival			
Age	≥68 yr	1.44 (1.02–2.04)	0.039
Lymphatic invasion	Present	3.40 (1.31–8.82)	0.012
Metastasis	Present	2.27 (1.26–4.07)	0.006

HR, hazard ratio.

## Discussion

This study showed that high protein expression of FATP4, CD36, and ACSL1 is associated with poor prognostic factors of NMIBC such as high grade, advanced stage, non-papillary growth type, and the presence of metastasis. Further, high FATP4 was an independent factor predicting short OS in NMIBC patients. Thus, upregulation of FATP4, CD36, and ACSL1 might contribute to NMIBC progression, which could be therapeutically targeted. Furthermore, patients with NMIBC that demonstrates high expression of these fatty acid transporters, need to be closely monitored and treated more aggressively.

Fatty acid uptake from extracellular sources is one of the mechanisms through which cancer cells adapt to the changing environment. When cancer cells confront metabolic stress such as hypoxia or attempt to change into a migratory phenotype, they depend on exogenous lipid uptake [16, 17]. In addition, as essential fatty acids such as linoleic acid cannot be naturally synthesized in the human body, they have to be taken up from dietary sources with the help of fatty acid transporters such as FATP. FATPs facilitate uptake of fatty acids by forming CoA-thioesters and trapping them inside the cell, though FATP can act as a membrane transporter for long chain fatty acids [18]. FATP4 is broadly distributed in the heart, liver, brain, kidney, muscle, skin, and endothelial cells, and is detectable on the plasma membrane and in the endoplasmic reticulum [19–21]. In this study, FATP4 was mainly found to be localized to the cytoplasm of bladder tumor cells. This finding suggests the possibility that FATP4 might function as a cytoplasmic enzyme rather than as a membrane transporter in bladder cancers. Consistent with previous reports regarding FATP4 expression in other cancers, FATP4 overexpression in this study was associated with poor prognostic factors of NMIBC and was an independent factor predicting short OS [11, 12]. Thus, as potent inhibitors against FATP4 such as 4-aryl-dihydropyrimidinones have already been identified, high FATP4 expression might be clinically exploited in risk stratification and in the targeted therapy of NMIBC patients [22].

In addition to FATP4, high expression of CD36 and ACSL1 in this study showed an association with unfavorable prognostic factors of NMIBC. CD36 is a multifunctional transmembrane glycoprotein receptor for various proteins including long chain fatty acids [23]. CD36 has been known to be associated with altered lipid metabolism and initiation of metastasis, contributing to cancer progression [9, 10]. Similar to FATP4, ACSL1 belongs to a family of long-chain fatty acyl CoA synthetases, which esterifies fatty acids with coenzyme A [24]. However, the prognostic value of ACSL1 has been differently described according to the cancer type and its protein expression in bladder cancer has never been studied. Although CD36 and ACSL1 failed to predict OS and RFS in this NMIBC cohort, it could be assumed that CD36 and ACSL1 might have roles in the progression of bladder cancers and could be targetable along with FATP4. Although FATP4, CD36, and ACSL1 showed weak positive correlation among them in this study, it is uncertain if FATP4, CD36 or ACSL1 might interact with each other and have a synergistic effect on the transport of fatty acids in bladder cancer cells.

In conclusion, this study identified that overexpression of FATP4, CD36, and ACSL1 might promote clinical progression of NMIBC, which could be exploited in risk stratification and in the targeted therapy of NMIBC patients. However, additional studies are required to understand the functional mechanisms of these fatty acid transporters in bladder cancer.

## Data Availability

All datasets presented in this study are included in the article/Supplementary Material.
