# Supplementary material for: Upregulation of Fatty Acid Transporters is Associated With Tumor Progression in Non-Muscle-Invasive Bladder Cancer
Source: Pathol Oncol Res. 2021 Mar 30;27:594705. doi: 10.3389/pore.2021.594705 (PMC8262182; doi:10.3389/pore.2021.594705)
Supplement: Supplementary file 1 [file DataSheet1.docx]

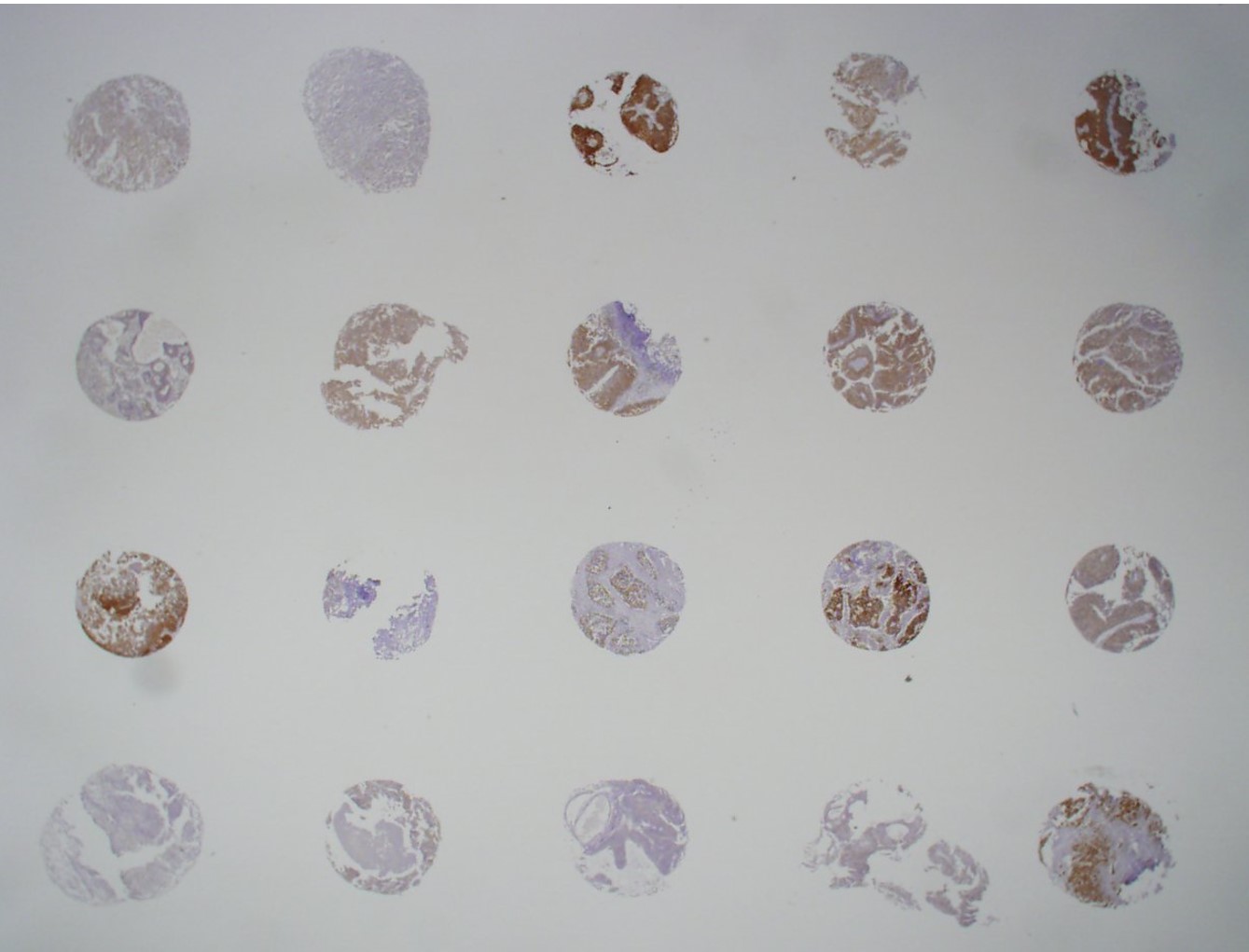


**Supplementary figure 1.** FATP4 expression in non-muscle-invasive bladder cancers. This figure of tissue microarray reveals a serial section of bladder cancer cases which were variously stained with FATP4 by immunohistochemistry (Original magnification, x12.5).


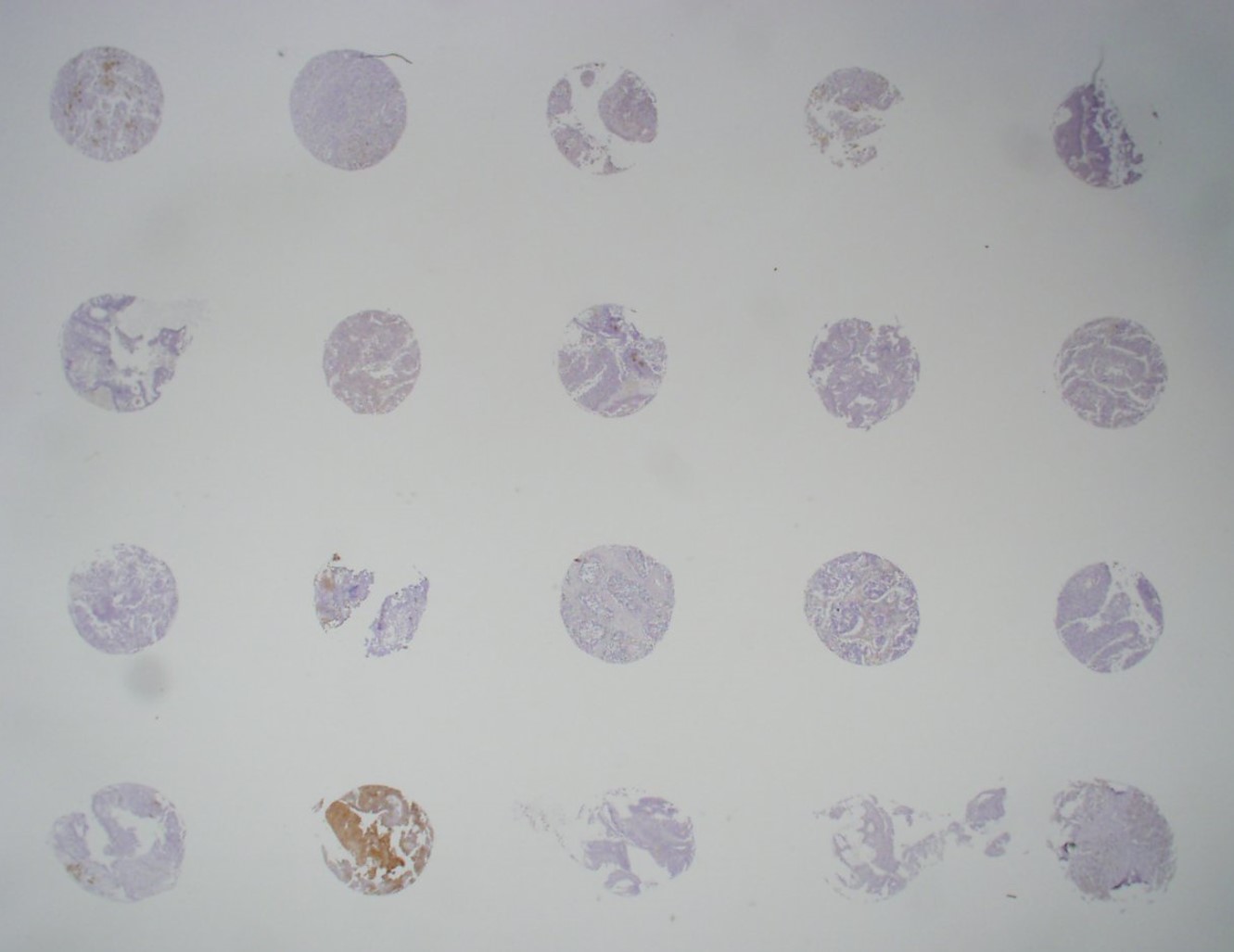


**Supplementary figure 2.** CD36 expression in non-muscle-invasive bladder cancers. This figure of tissue microarray reveals a serial section of bladder cancer cases which were variously stained with CD36 by immunohistochemistry (Original magnification, x12.5).


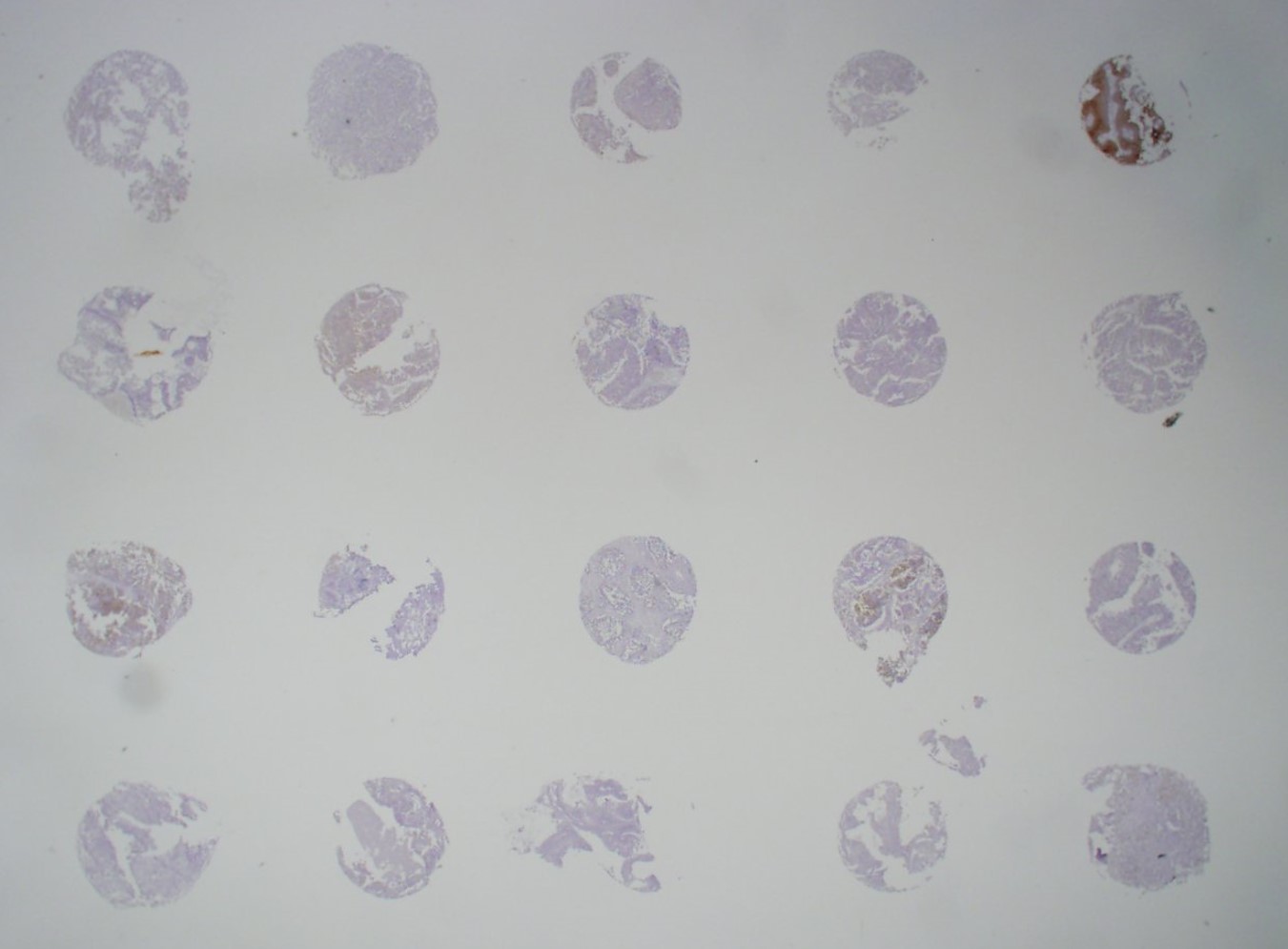


**Supplementary figure 3.** ACSL1 expression in non-muscle-invasive bladder cancers. This figure of tissue microarray reveals a serial section of bladder cancer cases which were variously stained with ACSL1 by immunohistochemistry (Original magnification, x12.5).


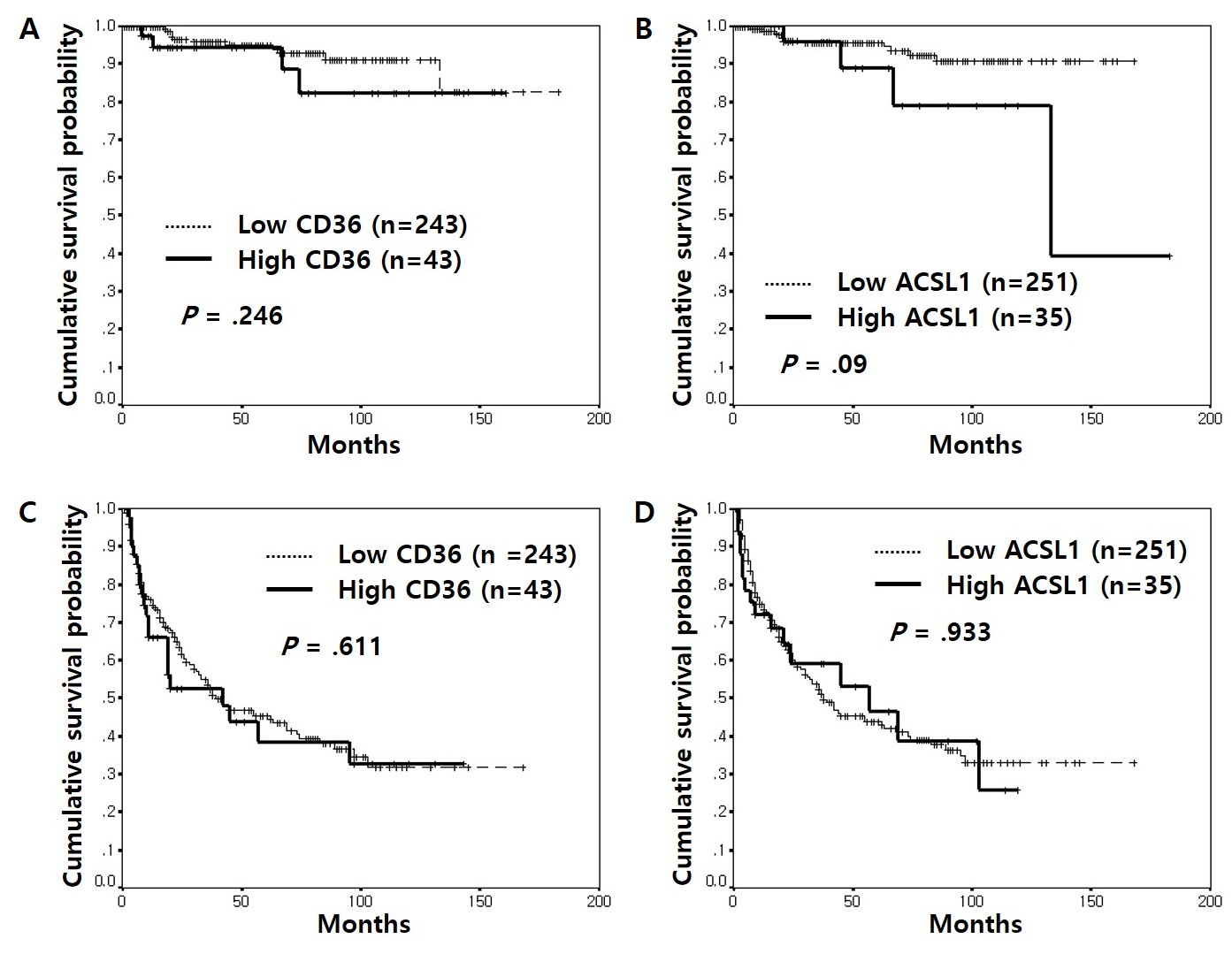


**Supplementary figure 4.** Kaplan-Meier survival analysis of CD36 and ACSL1 expressions in non-muscle-invasive bladder cancer. Both CD36 and ACSL1 expressions were not associated with overall (A and B) and recurrence-free survivals (C and D).
